# The Effect of Housing System on Disease Prevalence and Productive Lifespan of Dairy Herds—A Case Study

**DOI:** 10.3390/ani12131610

**Published:** 2022-06-22

**Authors:** Dorota Witkowska, Aneta Ponieważ

**Affiliations:** Department of Animal and Environmental Hygiene, University of Warmia and Mazury in Olsztyn, Oczapowski Street 5, 10-719 Olsztyn, Poland; poniewaz.aneta@wp.pl

**Keywords:** dairy cattle, housing system, diseases prevalence, lifespan, mastitis, limb disorder

## Abstract

**Simple Summary:**

Livestock welfare should be continuously improved to meet social and consumer expectations. Selected technological solutions can improve dairy cows’ welfare, which indirectly improves their health status and disease resistance. The aim of this study was to determine the effect of different housing systems on disease prevalence and the productive lifespan of dairy cows. The study was conducted on a single farm between 2015 and 2020. In total, 480 cows kept indoors in four different buildings using four housing systems were analyzed in each year of the study. The prevalence of the most common cattle diseases and the average productive lifespan of dairy cows were analyzed in each housing system, based on veterinary reports. The study demonstrated that housing system affects disease prevalence in dairy herds, but none of the tested solutions was without weaknesses. Deep litter was better overall. Lower morbidity in this system was associated with an increase in productive herd life, which was significantly longer. The prevalence of mastitis was reduced in the tie-stall barn, but the risk of lameness, retained placenta, parturient paresis and displaced abomasum was higher in this system. Overall morbidity was highest in the free-stall barns with a self-cleaning floor and with a slatted floor.

**Abstract:**

Selected technological solutions can impact health status of animals. The aim of this case study was to determine the effect of different housing systems on disease prevalence and the productive lifespan of dairy cows. In total, 480 cows kept indoors on one farm in four buildings using four different housing systems (a free-stall barn with a slatted floor; a free-stall barn with a self-cleaning floor; an open-pack barn with deep litter; a tie-stall barn with shallow litter) were analyzed. The data from 6 years, based on veterinary reports, were processed statistically in Statistica 13.00. The study demonstrated that the average productive lifespan was longer (*p* ≤ 0.01), by up to more than 8 months, in the system with deep litter, which was also characterized by the lowest disease prevalence (*p* ≤ 0.01), especially foot and some reproductive disorders. This trend was maintained in each year of the study period (2015–2020). In the tie-stall barn, the prevalence of mastitis was reduced, but the risk of lameness, retained placenta, parturient paresis and displaced abomasum was higher in this system (*p* ≤ 0.01). Overall morbidity was highest in the free-stall barns. Lower morbidity was associated with an increase in productive herd life.

## 1. Introduction

The global dairy cow population was estimated at 139 million head in December 2021. India is the world’s largest milk producer (58 million tons), followed by the EU countries (21 million tons) and Brazil (17 million tons) [1]. Poland is the fourth largest milk producer in the EU after Germany, France, the Netherlands and Italy [2]. According to OECD-FAO [3], global milk production is expected to increase by 1.7% annually between 2021 and 2030 and reach 1020 million tons in 2030.

Dairy farmers have to build new barns in order to increase their stocking rates. Housing conditions are one of the key factors that affect the health and welfare of dairy cows [4]. Farmers search for housing systems that, inter alia, optimize livestock health and maximize herd productivity. The popularity of free-stall barns has increased in the last 20 years. In Denmark, 70% of dairy cows were kept in tie-stall barns before 2000, whereas at present, the free-stall system is the most popular solution in newly built barns [5]. Research has demonstrated that loose housing improves animal health and welfare, and facilitates herd management [6,7].

The effectiveness of various housing systems is determined by herd size [6]. Large herds are more effectively managed in free-stall systems, which improve productivity by enhancing milk quality, increasing milk yields, decreasing energy consumption and labor intensity [5], and promoting the observance of environmental protection requirements [8]. In herds composed of more than 100 animals, total labor inputs can be even 30% lower in free-stall barns than in tie-stall housing systems. In farms with 70 dairy cows, a reduction in the labor inputs associated with feeding and cleaning operations in free-stall barns is counterbalanced by higher milking labor input [6]. In recent years, the popularity of free-stall housing systems has also increased in Polish dairy farms with more than 40 livestock units (LSU) [5].

A review of the literature comparing the effects of tie-stall and free-stall housing systems on the performance, health, fertility and behavior of dairy cows revealed that each system has its strengths and weaknesses, but the analyzed parameters tended to be higher in free-stall barns [9]. It is difficult to accept the lack of basic freedom of movement in tie-stall systems [10,11]. Research has shown that selected technological solutions can improve animal welfare and decrease greenhouse or toxic gas emissions and microbiological contamination in buildings, which indirectly improves the health status and disease resistance of animals [12,13].

The prevalence of diseases affecting dairy herds around the world is estimated at 25–68% [14], and a rising trend has been reported in recent years [15,16]. The increase in cattle morbidity [17] can be attributed to the growing productivity and intensification of livestock production. Intensive livestock production systems were introduced to cater to the growing demand for animal products and feed the world’s growing population [18]. Amory et al. [19] reported that lameness affects around 23% of cows in the EU, and the associated costs exceed EUR 1 billion each year. According to Willshire and Bell [20], the average treatment costs per animal reach around EUR 360 in a typical British herd. Most treatment and prevention costs constitute indirect costs associated with decreased milk yields, lower fertility, the higher prevalence of mastitis and, consequently, higher culling rates [11,21]. Diseases affecting dairy herds generate significant losses for breeders, which is why the risk of disease should be minimized at the stage of selecting the housing system.

Both free-stall and tie-stall housing systems have their advantages and weaknesses. According to Praks et al. [22], the risk of foot diseases is higher, whereas the prevalence of mastitis is considerably lower in free-stall than in tie-stall barns. Foot pain is easier to manage in the tie-stall system, but the expression of the cows’ natural behaviors is largely limited. Permanent tethering may have a negative effect on the behavior of the cows, such as the lack of comfortable resting surfaces, the absence of movement possibility and the lack of social interaction [10,11]. In comparison with tie-stall barns, loose housing systems are characterized by the absence of close contact between humans and animals, which can prevent accurate assessments of animal behavior or early symptoms of disease, thus contributing to prolonged treatment or even premature culling [11,23]. If the farm is using an AMS (automatic milking system), this limitation can be mitigated [24]. Moreover, individual feed intake is difficult to control in free-stall systems, and nutritional deficiencies can increase the risk of metabolic disorders and infertility [9].

Bovine hoof disorders can also compromise reproductive performance in dairy cows [25]. Lameness has a complex etiology which involves genetic factors, technical factors associated with the availability of the necessary equipment in barns, animal maintenance standards and nutrition. Hoof disorders pose a global problem [19]. According to estimates, approximately 10% of cows in large herds are culled due to hoof and foot diseases [26].

Mastitis is also one of the most prevalent and costly diseases that affects dairy producers worldwide [27]. In the United Kingdom, the annual prevalence of clinical mastitis has been estimated at 35% in the total dairy cow population. The costs associated with the treatment and prevention of mastitis in the British dairy sector exceed EUR 110 million each year. On average, every case of mastitis generates losses of EUR 200/cow [28]. The disease is also a major animal welfare problem that increases culling rates and is responsible for one in eight deaths in dairy herds [29,30].

The extent to which nutrition and genetic factors contribute to morbidity in dairy cows has been extensively researched. However, fewer studies have analyzed the role of environmental factors that have equally important implications for livestock welfare, which should be continuously improved to meet social and consumer expectations [10,31,32]. Animal welfare is vital consideration, especially since dairy herds are increasingly often kept indoors around the year. Lack of pasture access, limited mobility, various stressors, changes in behavior, rivalry within the herd and adverse environmental conditions compromise animal welfare and increase susceptibility to disease [22].

The following research hypothesis was formulated: the housing system influences the prevalence and type of diseases in dairy herds and affects productive herd life. Therefore, the aim of this case study was to determine the effect of different housing systems on disease prevalence and the productive lifespan of dairy cows.

## 2. Materials and Methods

The case study was conducted in one individual family farm between 2015 and 2020. In total, 480 Polish Holstein-Friesian Black and White (PHF-BW) cows kept indoors in four buildings with four different indoor housing systems were analyzed in each year of the study. The staff, management, feed source, nutrition strategy and approach to hygiene and disease prevention on the whole farm was the same. One hundred and twenty animals were monitored in every year of the study in each of the four buildings: (1) a free-stall barn with a slatted floor, (2) a free-stall barn with a self-cleaning floor, (3) an open-pack barn with deep litter and (4) a tie-stall barn with shallow litter.

- Free-stall barn with a slatted floor. The barn features pens where all cows can lie down at the same time. A cow pen is enclosed by metal bars, and it is a resting area that separates individual cows from the other animals in the barn. Each pen is laid with a rubber mat, and it is designed to hold one cow. The barn has a slatted floor which is scraped by robots.
- Free-stall barn with a self-cleaning floor. Animals have free access to pens with straw bedding. The barn has a solid floor which is cleaned automatically twice a day (before morning and evening milking).
- Open-pack barn with deep litter. Cows can choose warm and comfortable resting areas with deep litter bedding. Resting areas are not separated from manure areas, and they are supplemented with fresh and dry straw every day.
- Tie-stall barn. Each tie-stall features a resting area, a feeder, an automatic drinker and a tether that keeps cows inside individual stalls. Stalls are separated by rails. Each stall ends with an opening (with an estimated depth of 20 cm) where excrement is evacuated to the manure plate. Straw is replaced twice daily. In this system, cows are fed and milked individually. The floor is cleaned with a flap scraper. Cows have the possibility of exercising during milking (about 60 min), feeding (about 30 min), watering (about 60 min), manure removal (about 15 min every 2 h for 8 h) per day. Cow trainers were not used here.

**Figure 1:**
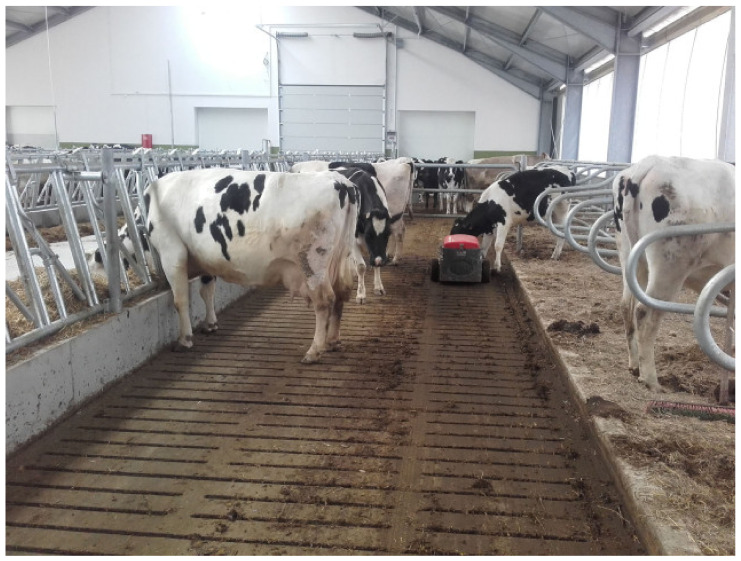
Free-stall barn with a slatted floor (A. Ponieważ).

**Figure 2:**
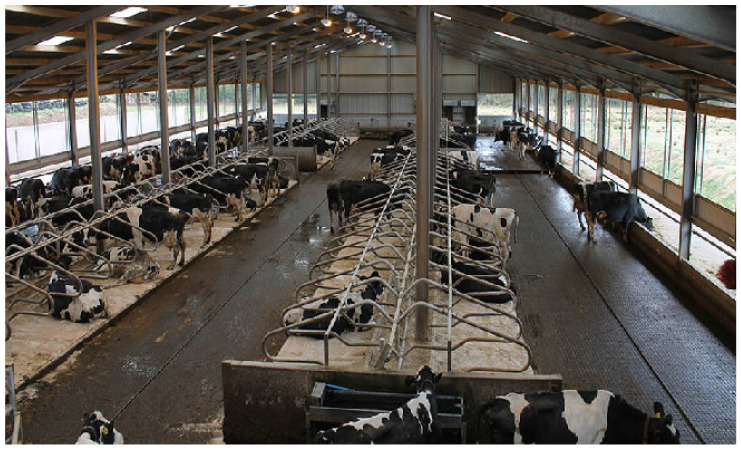
Free-stall barn with a self-cleaning floor (A. Ponieważ).

**Figure 3:**
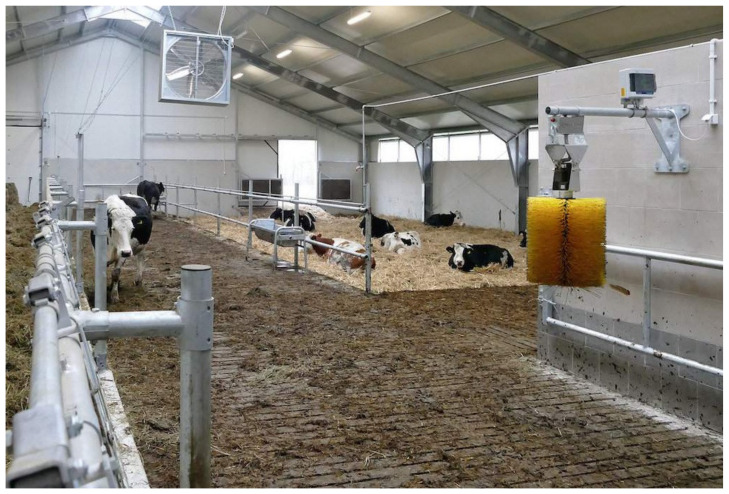
Open-pack barn with deep litter (A. Ponieważ).

**Figure 4:**
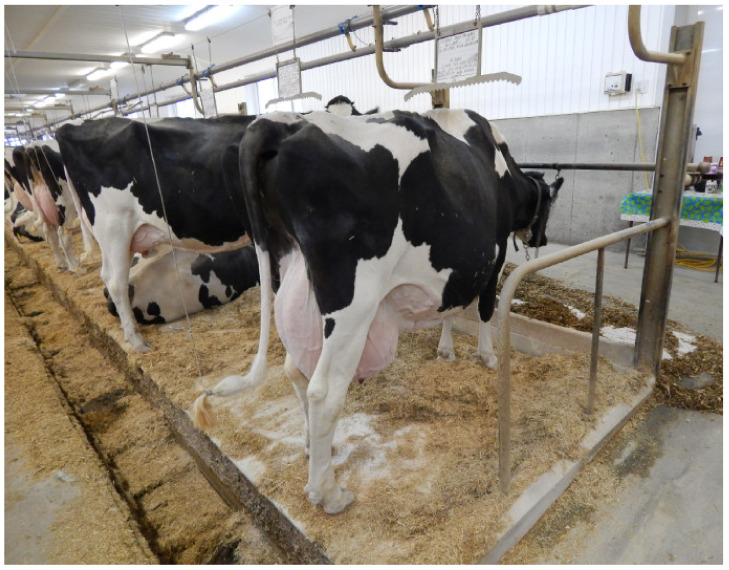
Tie-stall barn with shallow litter (A. Ponieważ).

The analyzed housing systems are described in detail in Table 1 and presented in Figure 1, Figure 2, Figure 3 and Figure 4.

Disease prevalence in the herd was monitored over a period of 6 years (2015–2020). The prevalence of the most common cattle diseases and the average productive lifespan of dairy cows were analyzed in each housing system, based on veterinary reports. The results were processed statistically in Statistica ver. 13.3 software (TIBCO Software Inc., Tulsa, OK). Data were tested for a normal distribution with the use of the Shapiro–Wilk test and one-way ANOVA. The significance of the differences between average morbidity (the rates of disease in a cow population) in the analyzed housing systems was determined by Tukey’s test.

## 3. Results

**Figure 5:**
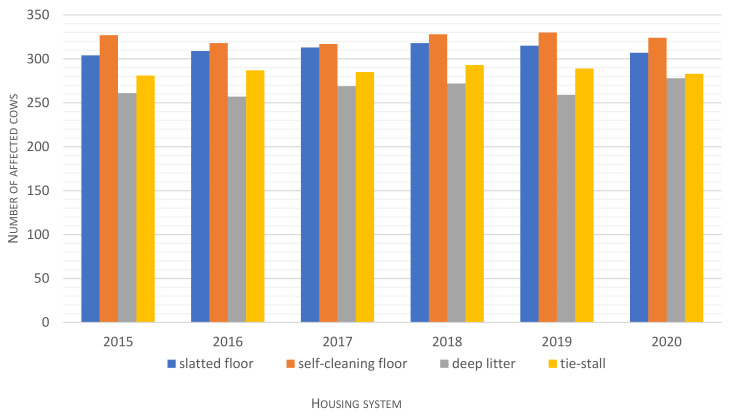
Average morbidity in the analyzed housing systems in each year of the study (2015–2020).

**Figure 6:**
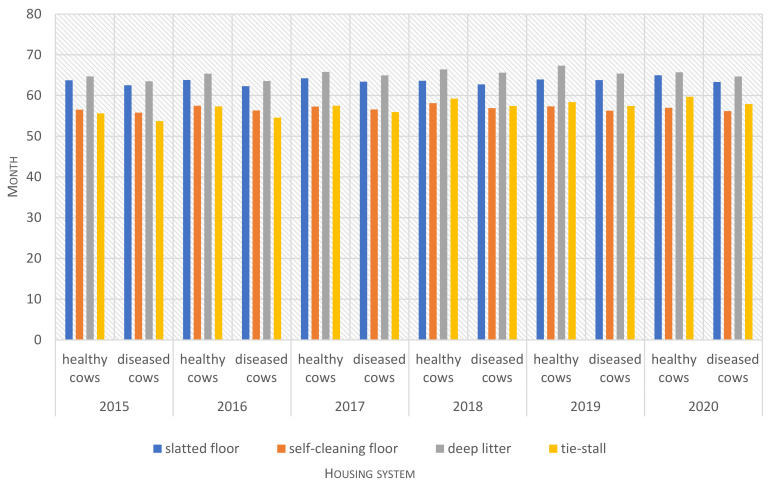
Productive lifespan (in months) of healthy and diseased cows kept in different housing systems in each year of the study (2015–2020).

During the 6-year study, disease prevalence was highest (*p* ≤ 0.01) in the free-stall housing system with a self-cleaning floor (Table 2). In this system, disease prevalence was 18% higher than in the open-pack system with deep litter, 12% higher than in the tie-stall system and 4% higher than in the free-stall system with a slatted floor. Morbidity was similar in each year of the analyzed period (2015–2020) (Figure 5). The average productive lifespan was longest in the system with deep litter, which was characterized by the lowest disease prevalence (*p* ≤ 0.01), and it was shortest in the free-stall system with a self-cleaning floor and the tie-stall system (Table 2). In these systems, productive herd life was more than 8 months shorter than in the deep litter system. This trend was maintained in each year of the study period (2015–2020) (Figure 6).

**Figure 7:**
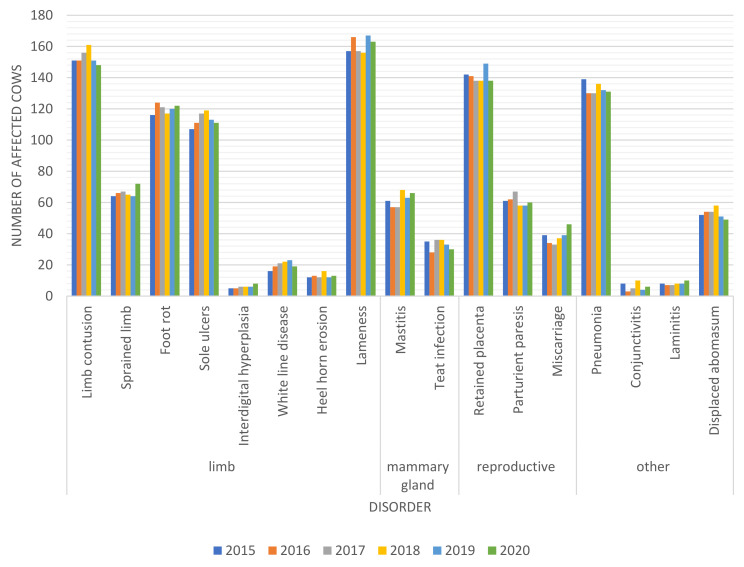
Prevalence of different diseases in the entire cow population in successive years of the study (2015–2020).

Foot diseases accompanied by lameness (mostly contusion, footrot and sole ulcers), retained placenta and pneumonia were most prevalent in the evaluated herds in each year (Figure 7) and during the entire period of the study (Table 3). Conjunctivitis, interdigital hyperplasia and laminitis were least frequently noted.

**Figure 8:**
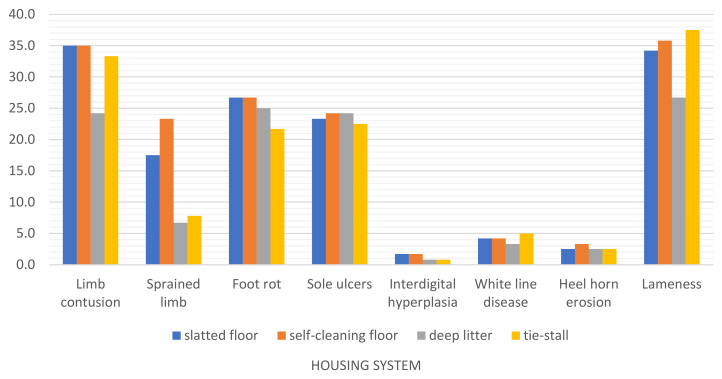
Prevalence of limb disorders in dairy cows kept in different housing systems in 2015–2020.

Foot disorders were least prevalent in the open-pack system with deep litter (Table 4, Figure 8), where the average prevalence of limb contusions, sprained limbs and white line disease was significantly lower (*p* ≤ 0.01). In turn, footrot was significantly less prevalent in the tie-stall system in comparison with the remaining systems (*p* ≤ 0.01), and sprained limbs were also noted significantly less frequently in the tie-stall system (*p* ≤ 0.01) than in free-stall barns with self-cleaning and slatted floors. However, the tie-stall system was characterized by a higher prevalence of lameness in comparison with the remaining systems *(p* ≤ 0.01). The highest number of limb contusions was noted in free-stall barns with slatted and self-cleaning floors (*p* ≤ 0.01).

**Figure 9:**
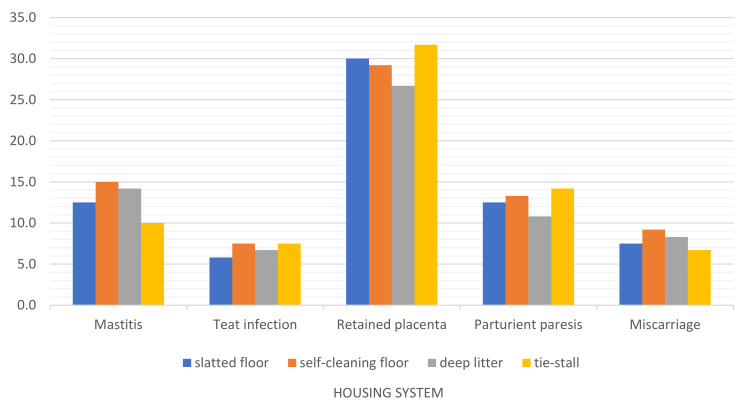
Prevalence of mammary gland and reproductive disorders in dairy cows kept in different housing systems in 2015–2020.

An analysis of the average morbidity (Table 5) and prevalence (Figure 9) of mastitis and reproductive disorders in different housing systems indicates that the risk of retained placenta and parturient paresis was significantly lower on deep litter (*p* ≤ 0.01). Retained placenta (the most common reproductive disorder in this study) was significantly more prevalent in the tie-stall system and in the free-stall barn with a slatted floor (*p* ≤ 0.01). In turn, the tie-stall system was characterized by a lower risk of mastitis than the open-pack barns with deep litter and a self-cleaning floor (*p* ≤ 0.01). Mastitis was also less prevalent in the barn with a slatted floor than in systems with deep litter and a self-cleaning floor, although the noted differences were not statistically significant. No differences in morbidity linked to teat infections were observed among groups. The number of miscarriages was significantly higher in herds kept on a self-cleaning floor than in the tie-stall system (*p* ≤ 0.01).

**Figure 10:**
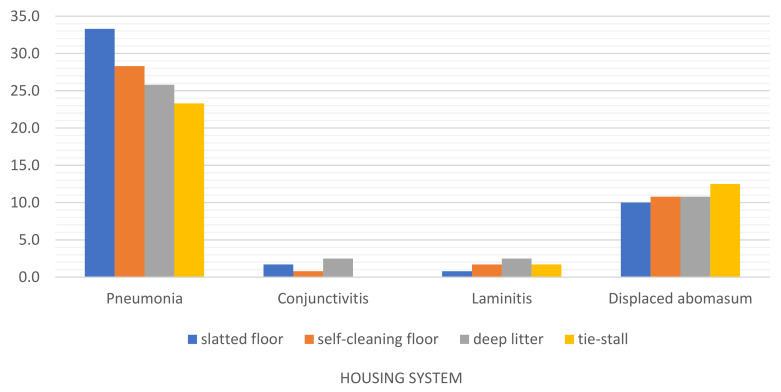
Prevalence of the remaining diseases in dairy cows kept in different housing systems in 2015–2020.

In the group of the remaining diseases (Table 6, Figure 10), pneumonia was relatively frequently noted, particularly in the barn with a slatted floor (*p* ≤ 0.01). This disease was least prevalent in the tie-stall system (*p* ≤ 0.01). The tie-stall system was associated with a higher risk of displaced abomasum (*p* ≤ 0.01), but unlike the remaining systems, not a single case of conjunctivitis was noted in tethered cows. Cows kept on deep litter were significantly more often (*p* ≤ 0.01) affected by conjunctivitis and laminitis, which were least prevalent in the barn with a slatted floor (*p* ≤ 0.01).

## 4. Discussion

Bovine hoof disorders were noted in 56% of the studied cow population. Their prevalence remained within the global average (30–70%) [14,15]; nevertheless, it was definitely excessive. By comparison, in the Netherlands, an annual prevalence rate of 30% (clinical cases) is not acceptable, solely from the ethical point of view [33]. The health status of the investigated herds could be improved by providing cows with access to pastures in summer and paddocks in winter. Physical activity, fresh air and exposure to sunlight promote locomotive health in cattle [15,16,17,18,19,20,21,34].

It should be emphasized that in this case study, we compared four buildings using four different indoor housing systems on one farm. Only fully indoor farm was considered in this study. There is high between-farm variation even if they use the same system, so one farm cannot be used to generalize about an entire system or compared with systems where the herds have access to pasture. The results of this case study clearly indicate that dairy cows housed in tie-stall and free-stall barns with a self-cleaning floor, a slatted floor or on deep litter are commonly affected by foot (in particular hoof) disorders, mastitis, reproductive and respiratory diseases (Table 3, Figure 6). The open-pack system with deep litter was characterized by the lowest disease prevalence and the longest productive herd life (Table 2, Figure 5). In this system, animals can freely move, have sufficient resting area and can spend most of the day lying down. Absorbent and well-maintained straw litter creates a natural resting area, acts as a thermal insulator and prevents slipping [35]. It should be underlined that cows’ ability to perform natural behaviors and affective states are as relevant, if not more, in assessing their overall welfare [36]. The free-stall system with a self-cleaning floor was least conducive to animal welfare. In this system, disease prevalence was highest and herd life was more than 8 months shorter than in the barn with deep litter. As shown in Figure 2, cows housed on a self-cleaning floor have free access to a resting area covered with shallow straw litter. Shallow litter has lower absorbance and insulating properties, which can contribute to inflammation. Additionally, a solid floor does not provide shock absorption and compromises limb stability [37]. Productive herd life significantly affects the profitability of milk production [38,39], and it should not be shorter than 5–8 lactation cycles [40]. In recent years, the productive lifespan of many herds has been reduced to three lactations. This period is too short to enable the effective selection of mother cows, and it should be prolonged to five lactations. Productivity in the first lactation is an important consideration in view of the decreasing lifespan of dairy herds [9].

According to Loberg et al. [41] and Keil et al. [42], free-stall barns are characterized by a higher prevalence of foot disorders but a much lower risk of mastitis. In the present study, the types and prevalence of disease were significantly influenced by the housing system. The prevalence of limb disorders such as contusions, sprains and white line disease was lowest (*p* ≤ 0.01) on deep litter (Table 4, Figure 8). Soft straw litter prevents cows from tripping and slipping, and it protects hoofs against excessive wear [35]. The prevalence of contusions and sprains was highest on slatted and self-cleaning floors (*p* ≤ 0.01). Slatted floors and cracked concrete floors increase the risk of tripping, slipping and limb injuries. The tie-stall system where the animals’ range of motion is limited by a chain tether was also characterized by a significantly higher prevalence of limb contusions, sprains and white line disease (*p* ≤ 0.01) in comparison with deep litter. Prolonged standing significantly contributes to limb disorders in cattle. A large body mass exerts a considerable load on the joints and often leads to hoof horn abnormalities [43], which is why limb disorders were more prevalent in the tie-stall system.

In turn, footrot (Table 4, Figure 8) was least frequently noted (*p* ≤ 0.01) in the tie-stall barn where the risk of sole ulcers was also considerably lower than in other systems (although the difference was not statistically significant). These results indicate that regular manure removal with a flap scraper eliminates excessive moisture and prevents hoof softening. Cows that do not come into direct contact with manure are also less exposed to bacteria and other pathogens that cause infections [44,45]. In housing systems with self-cleaning and slatted floors, frequent contact with hard surfaces covered with manure and mud promotes excessive hoof wear and softening [33]. Uneven surfaces, slippery floors and poor hygiene exacerbate these risks. Wet floors with accumulated manure also increase exposure to the pathogens that cause foot and hoof diseases [46,47]. For this reason, footrot was most prevalent on deep litter and in other free-stall systems where moisture tended to accumulate on the floor (self-cleaning floor, Figure 2) and where manure was collected under a slatted floor (Figure 1). Footrot and sole ulcers are relatively common problems in Polish dairy farms [48]. In a study of the British dairy sector conducted by Bell [49], sole ulcers were identified as the most prevalent foot disease in the past, but in the following years, around of 25% of all diagnosed cases of lameness were associated with bacterial infections, including infections caused by anaerobic bacteria. Most limb disorders result from poor maintenance [33], which is why prevention should begin at the stage of designing and equipping dairy barns. Effective herd management, healthy microclimate conditions in barns, regular litter replacement with high-quality straw, spacious stalls that enable free movement and provide comfortable resting areas, elimination of architectural barriers (rational installation and operation of barn equipment, particularly manure scrapers), sloping floors that effectively drain slurry, slip-resistant floors, and the separation of feed and water stations from resting areas decrease the risk of injury and improve barn hygiene [50]. Early recognition of disease symptoms shortens and facilitates treatment, which is why the health status of dairy herds should be monitored daily. Regular hoof care and trimming by a professional are also an important part of the prevention strategy to combat serious foot problems. Physical activity promotes locomotive health in dairy herds [51,52,53]. Disinfectant foot baths involving shallow pools, mats and foam treatments are effective in preventing and treating selected foot diseases [54]. In this study, the prevalence of heel horn erosion, white line disease and interdigital hyperplasia did not exceed 5%. Interdigital dermatitis was far more frequently reported in other studies [14,55,56,57]. According to Enevoldsen [58], in comparison with other foot diseases, heel horn erosion is most significantly affected by the conditions inside dairy barns and herd maintenance standards. In the present study, cows had limited access to paddocks or runs, which could have contributed to the risk of heel horn erosion.

According to a report of the European Food Safety Agency [59], the most important risk factor for mastitis is poor bedding hygiene and contamination, particularly by environmental pathogens. Straw and manure are colonized by opportunistic pathogens such as *E. coli*, *S. uberis*, *S. faecalis* and *Klebsiella* spp., which mostly infect the mammary gland [29]. Udders can be constantly exposed to contaminated straw, which enables pathogenic microorganisms to enter the mammary gland and initiate the infection [60]. In this study, the number of teat infections did not differ statistically among the compared systems, and mastitis was least prevalent in the tie-stall system, in which cows have less direct contact with wet or contaminated straw (Table 5, Figure 9).

Miscarriage was most prevalent in the system with a self-cleaning floor and least prevalent in the tie-stall barn. In the tie-stall system, pregnant cows are less likely to slip, trip or be pushed by other animals because they do not have to compete for position at the feeding station. Unlike in loose housing systems, there is no risk that a scared animal will bolt and injure a resting cow, which can lead to udder and teat damage or even miscarriage. Tethered cows can also be more effectively monitored for overall health and feed intake.

Retained placenta was the most common problem in the tie-stall system, and it was also significantly more frequent (*p* ≤ 0.01) in the free-stall barn with a slatted floor than in the barn with deep litter (Table 5, Figure 9). Tethered cows are unable to move freely, and their resting areas are less comfortable. Due to limited space, the cow is unable to assume the correct position during labor and veterinary assistance is difficult, which can increase the risk of retained placenta. In contrast, deep litter offers a much more comfortable environment during labor, and the animal can be easily accessed by a veterinarian. The open-pack barn with deep litter was also characterized by the lowest prevalence of parturient paresis. This disorder affects 5–10% and even up to 30% of dairy herds [61]. According to Wang [62], parturient paresis can increase the risk of retained placenta or displaced abomasum, which was confirmed by this study. Displaced abomasum was more prevalent in the tie-stall system (Table 6, Figure 10) because restricted mobility can disrupt gastric peristalsis and lead to displacement of the abomasum. Tethering also impairs feed intake, which can contribute to gastrointestinal disorders.

Laminitis was noted sporadically (Table 3), and it was slightly prevalent in cows kept on deep litter in comparison with the barn with a slatted floor (Table 6, Figure 10) The occurrence of laminitis can be reduced by regularly removing manure, supplying fresh straw, cleaning and trimming hooves (on deep straw, hooves are not trimmed through natural abrasion) [63]. Mastitis can also contribute to laminitis because bacterial endotoxins disrupt blood circulation in the hoof capsule [64,65]. Laminitis is also a metabolic disorder whose risk increases in the presence of comorbidities such as ketosis, acidosis and alkalosis. However, these disorders were not observed in this study.

Pneumonia was relatively frequently noted in the analyzed herds (Table 6, Figure 10), particularly in the free-stall housing system with a slatted floor (*p* ≤ 0.01). This disease was least prevalent in the tie-stall system (*p* ≤ 0.01). Pneumonia can be caused by bacterial, viral, fungal or parasitic (nematode) infections. Stress, poor hygiene, temperature fluctuations, high humidity and inadequate ventilation in the barn can compromise immunity and increase susceptibility to pneumonia [66]. Barns with a slatted floor are characterized by high concentrations of toxic gases, including ammonia, which also contribute to respiratory infections [13,67,68]. This observation could explain the higher prevalence of pneumonia in cows kept on a slatted floor. Ammonia is also a frequent cause of conjunctivitis, and the prevalence of this disease was higher in cows housed on a slatted floor and on deep litter. Manure tends to accumulate on slatted floors and deep litter, which explains the higher ammonia emissions in these housing systems [13].

## 5. Conclusions

This case study demonstrated that the housing system affects disease prevalence in dairy herds, and none of the tested solutions was without weaknesses. In terms of health status, the friendliest for cows is the open-pack system with deep litter. The average productive lifespan was longest by more than 8 months in case of this solution. Deep litter also reduced the risk of disease prevalence, especially foot and some reproductive disorders, but it was not associated with a decreased risk of mastitis. The prevalence of mastitis was reduced in the tie-stall barn, but the risk of lameness, retained placenta, parturient paresis and displaced abomasum was higher in this system. Overall morbidity was highest in the free-stall barns with a self-cleaning floor and with a slatted floor, in which herds had considerably increased disease prevalence, mainly the risk of foot diseases and pneumonia. Higher morbidity was associated with a decrease in productive herd life. This problem can be minimized by gradually eliminating the key factors that contribute to limb diseases. Solutions that compromise animal welfare should be eliminated at the stage of designing cattle barns.

## Figures and Tables

**Table 1:** Characteristics of the evaluated housing systems.

	Free-Stall Systems			Tie-Stall System
	Slatted Floor	Self-Cleaning Floor	Deep Litter	
**Herd size (head)**	**120**	**120**	**120**	**120**
**Floor type**	Slatted	Solid	Straw	Solid concrete
**Bedding**	Rubber mat with a thickness of 6 cm	Straw	Straw	Straw
**Manure removal**	Manure is removed by robots every 240 min	Manure is scraped twice a day before each milking	Fresh straw is supplied each day; manure is removed four times a year	Manure is removed twice a day with a flap scraper before every milking
**Ventilation**	Natural ventilation	Natural ventilation	Mechanical ventilation	Natural and mechanical ventilation
**Milking system**	Parallel milking parlor	Fishbone milking parlor	Fishbone milking parlor	Pipeline milking machine
**Hoof care**	Foot bath: formalin and copper sulfate, twice a month	Foot bath: formalin and copper sulfate, twice a month	Foot bath: formalin and copper sulfate, twice a month	Manual sprayer: formalin and copper sulfate, twice a month
**Hoof trimming**	Twice a year and if required	Twice a year and if required	Twice a year and if required	If required
**Feed pushing**	Feed pusher robot	Feed pusher robot	Feed pusher robot	Feed pusher robot
**Feed pushing frequency**	At intervals of 180 min	At intervals of 210 min	At intervals of 180 min	At intervals of 210 min
**Feed**	PMR+ feeding station	PMR+ feeding station	PMR+ feeding station	TMR
**Control of estrus and feed intake**	24 h electronic monitoring system	24 h electronic monitoring system	24 h electronic monitoring system	Observation

**Table 2:** Overall (Σ) and average (±SEM; minimum–maximum) morbidity in the studied population of dairy cows and productive herd life (in months) in different housing systems in 2015–2020.

Parameter	Total	Housing System				*p*-Value
		Slatted Floor	Self-Cleaning Floor	Deep Litter	Tie-Stall	
**Overall morbidity**	7124	1866	1944	1596	1718	-
**Average morbidity**	296.83 ± 4.80 257.00–330.00	311.02 ^B^ ± 2.14 304.00–318.00	324.14 ^A^ ± 2.20 317.00–330.00	266.00 ^D^ ± 3.37 257.00–278.00	286.33 ^C^ ± 1.76 281.00–293.00	0.000177
**Average productive lifespan of the entire population**	60.66 ± 0.57 53.72–67.32	63.53 ^B^ ± 0.22 62.28–64.97	56.81 ^C^ ± 0.19 55.78–58.11	65.25 ^A^ ± 0.31 63.50–67.32	57.06 ^C^ ± 0.52 53.72–59.68	0.000169
**Average productive lifespan of healthy cows**	61.29 ± 0.79 55.62–67.32	64.05 ^B^ ± 0.20 63.61–64.97	57.28 ^C^ ± 0.22 56.51–58.11	65.88 ^A,B^ ± 0.37 64.68–67.32	57.95 ^C^ ± 0.60 55.62–59.68	0.000175
**Average productive lifespan of diseased cows**	60.03 ± 0.82 53.72–65.61	63.00 ^A^ ± 0.24 62.28–63.78	56.34 ^B^ ± 0.15 55.78–56.89	64.61 ^A^ ± 0.37 63.50–65.61	56.17 ^B^ ± 0.70 53.72–57.89	0.000175

**^A,B,C^**—mean values in rows differ significantly at *p* ≤ 0.01.

**Table 3:** Overall and (Σ) and average (±SEM) morbidity and the prevalence (%) of different diseases or symptoms in the entire cow population in 2015–2020.

Disorders		Total	Mean	SEM	%
**Limb**	**Limb contusion**	918	38.25	0.12	31.88
	**Sprained limb**	398	16.58	0.18	13.82
	**Footrot**	720	30.00	0.06	25.00
	**Sole ulcers**	678	28.25	0.03	23.54
	**Interdigital hyperplasia**	36	1.50	0.01	1.25
	**White line disease**	120	5.00	0.02	4.17
	**Heel horn erosion**	78	3.25	0.02	2.71
	**Lameness**	966	40.25	0.11	33.54
**Mammary gland**	**Mastitis**	372	15.50	0.06	12.92
	**Teat infection**	198	8.25	0.03	6.88
**Reproductive**	**Retained placenta**	846	35.25	0.06	29.38
	**Parturient paresis**	336	15.25	0.04	12.71
	**Miscarriage**	228	9.50	0.04	7.92
**Remaining**	**Pneumonia**	798	33.25	0.10	27.71
	**Conjunctivitis**	36	1.50	0.02	1.25
	**Laminitis**	48	2.00	0.02	1.67
	**Displaced abomasum**	318	13.25	0.03	11.04
	**Total**	**7124**	**17.46**	**0.04**	**14.55**

*n* (total number of cows) = 2880.

**Table 4:** Limb disorders affecting dairy cows kept in different housing systems in 2015–2020 (mean ± SEM).

Disorder or Symptom	Housing System				*p*-Value	Total
	Slatted Floor	Self-Cleaning Floor	Deep Litter	Tie-Stall		
**Limb contusion**	42.00 ^A^ ± 1.09	42.00 ^A^ ± 0.57	29.00 ^B^ ± 0.57	40.00 ^A^ ± 0.63	≤0.00001	38.25 ± 1.17
**Sprained limb**	21.00 ^B^ ± 0.57	28.00 ^A^ ± 0.96	8.00 ^C^ ± 0.36	9.33 ^C^ ± 0.42	≤0.00001	16.58 ± 1.75
**Footrot**	32.00 ^A^ ± 0.96	32.00 ^A^ ± 0.85	30.00 ^A^ ± 0.44	26.00 ^B^ ± 0.51	0.000024	30.00 ± 0.61
**Sole ulcers**	28.00 ± 0.96	29.00 ± 0.68	29.00 ± 0.63	27.00 ± 0.57	0.195323	28.25 ± 0.38
**Interdigital hyperplasia**	2.00 ± 0.25	2.00 ± 0.25	1.00 ± 0.25	1.00 ± 0.25	0.095101	1.50 ± 0.15
**White line disease**	5.00 ± 0.51	5.00 ± 0.36	4.00 ^B^ ± 0.25	6.00 ^A^ ± 0.51	0.030488	5.00 ± 0.24
**Heel horn erosion**	3.00 ± 0.36	4.00 ± 0.36	3.00 ± 0.36	3.00 ± 0.51	0.245052	3.25 ± 0.21
**Lameness**	41.00 ^B^ ± 0.89	43.00 ^A,B^ ± 1.06	32.00 ^C^ ± 0.68	45.00 ^A^ ± 0.57	≤0.00001	40.25 ± 1.10

*n* = 6 (number of cases in each year of the study (2015–2020); **^A,B,C^**: mean values in rows differ significantly at *p* ≤ 0.01.

**Table 5:** Mammary gland and reproductive disorders in dairy cows kept in different housing systems in 2015–2020 (mean ± SEM).

Disorder or Symptom	Housing System				*p*-Value	Total
	Slatted Floor	Self-Cleaning Floor	Deep Litter	Tie-Stall		
**Mastitis**	**15.00 ± 0.57**	18.00 ^A^ ± 0.81	17.00 ^A^ ± 0.73	12.00 ^B^ ± 0.97	0.000145	15.50 ± 0.60
**Teat infection**	7.00 ± 0.57	9.00 ± 0.68	8.00 ± 0.63	9.00 ± 0.73	0.130295	8.25 ± 0.35
**Retained placenta**	36.00 ^A^ ± 1.57	35.00 ± 0.81	32.00 ^B^ ± 0.36	38.00 ^A^ ± 0.77	0.003085	35.25 ± 0.64
**Parturient paresis**	15.00 ± 0.57	16.00 ^A^ ± 0.36	13.00 ^B^ ± 0.51	17.00 ^A^ ± 0.57	0.000182	15.25 ± 0.39
**Miscarriage**	9.00 ± 1.06	11.00 ^A^ ± 0.51	10.00 ± 0.77	8.00 ^B^ ± 0.36	0.048768	9.50 ± 0.41

*n* = 6 (number of cases in each year of the study (2015–2020); **^A,B,C^**: means in rows differ significantly at *p* ≤ 0.01.

**Table 6:** Other diseases affecting dairy cows kept in different housing systems in 2015–2020 (mean ± SEM).

Disease	Housing System				*p*-Value	Total
	Slatted Floor	Self-Cleaning Floor	Deep Litter	Tie-Stall		
**Conjunctivitis**	2.00 ^A^ ± 0.51	1.00 ^B^ ± 0.36	3.00 ^A^ ± 0.36	0.00 ^B^ ± 0.00	0.000079	1.50 ± 0.28
**Pneumonia**	40.00 ^A^ ± 1.23	34.00 ^B^ ± 0.51	31.00 ^C^ ± 0.32	28.00 ^D^ ± 0.32	≤0.00001	33.25 ± 0.98
**Laminitis**	1.00 ^B^ ± 0.25	2.00 ± 0.25	3.00 ^A^ ± 0.36	2.00 ± 0.33	0.002667	2.00 ± 0.20
**Displaced abomasum**	12.00 ^B^ ± 0.63	13.00 ± 0.51	13.00 ± 0.51	15.00 ^A^ ± 0.31	0.004565	13.25 ± 0.33

*n* = 6 (number of cases in each year of the study (2015–2020); **^A,B,C^**: mean values in row differ significantly at *p* ≤ 0.01.

## Data Availability

Not applicable.
